# Medical Society of Tennessee

**Published:** 1840

**Authors:** 


### MEDICAL SOCIETY OF TENNESSEE.

We had the pleasure of being present at the meeting of the Medical Society of Tennessee, in Nashville, on the first Monday in May—the ***eleventh annual*** meeting of the Society, which, in keeping them up for so many years without interruption, has manifested a commendable persistency but too rare in such associations. The Society was chartered in the fall of 1829, and in the following May a large proportion of the members elected assembled in Nashville. It was the first united public effort in the State at improving the character and standing of the profession, and the meeting was large and spirited. Every quarter of the commonwealth had its representatives, and the assemblage embraced as large an amount of professional talent as we have ever seen brought together; but owing to a defect in the charter, which succeeding Legislatures have refused to supply, the interest of many members in the Society has abated, and its subsequent meetings have been less numerously attended. The law establishing the Society povided for the appointment of a Board of Censors for East, Middle, and West Tennessee, and made it the duty of these Censors to examine all candidates for the practice of physic who should make application to them; but it did not make it obligatory upon candidates to pass such an examination before engaging in the practice. It was easy to forsee that few aspirants to practice, under such a provision, would trouble the Censors to inquire into their qualifications. The hope that the Legislature would see the policy of making this salutary requisition, has hitherto been disappointed, and as the measure would not probably be a *popular* one, it is not likely soon to be adopted. Regarding this, which was the fact with many of the original members, as the primary and far the most important object of the Society they have despaired of its usefulness, and ceased to attend its meetings.

This, it has always been our opinion, is a great mistake. The regulation of medical practice by law, is not the most important function of such associations. A much higher object is to extend and improve professional knowledge—to multiply and perfect our remedies and their application—to develope the causes of our endemic diseases, and devise the measures to prevent or relieve them—to awaken a spirit of inquiry, and promote friendly intercourse among the members of the profession. In regard to all these objects, the Medical Society of Tennessee has already exerted a good influence, and is capable of extending it indefinitely hereafter. It will, if continued and duly supported, soften those asperities which are felt to be among the greatest evils of a medical life, and which have been the standing reproach of our profession. It will excite and keep alive a spirit of emulation and research, and bring to light the results of much valuable experience. The publication of its transactions will give character to the faculty of the State abroad, as they have already made it favorably known. The soil and climate of the state, the situation and exposure of the localities particularly subject to disease, the minerals, water, winds, indigenous medicinal plants, the endemic and epidemic disorders—all these will come under the investigation of the Society, and whatever else in diet and modes of living, or in moral and intellectual culture, contributes to health, or engenders disease. It is able to do the state and the profession much service, and it was with great pleasure, therefore, that we heard the members at the last meeting unanimously resolve, that they would not willingly let the Society die.

Dr. Robertson, perhaps the oldest, and one of the most eminent practitioners in the State, having declined to serve any longer as President, Dr. Hogg was unanimously elected to that office for the next ensuing two years. The pleasure of conferring this well-merited honor upon the venerable member, was greatly enhanced with every individual present by the circumstance, that he had just resumed the duties of his profession after a protracted illness—an affection of the lungs—which it was long feared would prove fatal. It was made the duty of the President to deliver an address before the society at the next annual meeting, and one may be expected from Dr. Hogg to which his great experience will impart a high value. Dr. Buchanan, of Columbia, was elected Vice President, and is required by the terms of his office to deliver an address at the meeting in May, 1842. Dr. B. is one of the most zealous and enterprising members of the Society, with an active mind, high attainments, and true professional ambition, and will be faithful and able in the discharge of that duty.

The other officers elected, were Dr. Waters, Corresponding Secretary; Dr. Martin, Recording Secretary; Dr. Shelby, Treasurer, and Dr. Yandell, Orator. The Board of Censors for the preceding year, were re-elected. A number of gentlemen were appointed to read papers on the topography and diseases of different regions of the State, a premium of 50 dollars was offered for the best Essay on Billious Fever, and, altogether, a large amount of literary exercise was provided for the next meeting. Y.

